# *DIRS1*-like retrotransposons are widely distributed among Decapoda and are particularly present in hydrothermal vent organisms

**DOI:** 10.1186/1471-2148-9-86

**Published:** 2009-04-28

**Authors:** Mathieu Piednoël, Eric Bonnivard

**Affiliations:** 1 UMR 7138 Systématique Adaptation Evolution, Equipe Génétique et Evolution, Université Pierre et Marie Curie Paris 6, Case 5, Bâtiment A, porte 427, 7 quai St Bernard, 75252 Paris Cedex 05, France

## Abstract

**Background:**

Transposable elements are major constituents of eukaryote genomes and have a great impact on genome structure and stability. Considering their mutational abilities, TEs can contribute to the genetic diversity and evolution of organisms. Knowledge of their distribution among several genomes is an essential condition to study their dynamics and to better understand their role in species evolution.* DIRS1*-like retrotransposons are a particular group of retrotransposons according to their mode of transposition that implies a tyrosine recombinase. To date, they have been described in a restricted number of species in comparison with the LTR retrotransposons. In this paper, we determine the distribution of *DIRS1*-like elements among 25 decapod species, 10 of them living in hydrothermal vents that correspond to particularly unstable environments.

**Results:**

Using PCR approaches, we have identified 15 new *DIRS1*-like families in 15 diverse decapod species (shrimps, lobsters, crabs and galatheid crabs). Hydrothermal organisms show a particularly great diversity of *DIRS1*-like elements with 5 families characterized among Alvinocarididae shrimps and 3 in the galatheid crab *Munidopsis recta*. Phylogenic analyses show that these elements are divergent toward the *DIRS1*-like families previously described in other crustaceans and arthropods and form a new clade called AlDIRS1. At larger scale, the distribution of *DIRS1*-like retrotransposons appears more or less patchy depending on the taxa considered. Indeed, a scattered distribution can be observed in the infraorder Brachyura whereas all the species tested in infraorders Caridea and Astacidea harbor some *DIRS1*-like elements.

**Conclusion:**

Our results lead to nearly double both the number of *DIRS1*-like elements described to date, and the number of species known to harbor these ones. In this study, we provide the first degenerate primers designed to look specifically for *DIRS1*-like retrotransposons. They allowed for revealing for the first time a widespread distribution of these elements among a large phylum, here the order Decapoda. They also suggest some peculiar features of these retrotransposons in hydrothermal organisms where a great diversity of elements is already observed. Finally, this paper constitutes the first essential step which allows for considering further studies based on the dynamics of the *DIRS1*-like retrotransposons among several genomes.

## Background

Deep-sea vents are chemosynthetic environments which are considered as extreme as compared to usual life standards due to their physico-chemical characteristics. For instance, they show high levels of temperature, pressure, heavy metals and sulfide [1]. They are also highly variable, with physico-chemical shifts occurring over very short spatial and temporal scales [2]. Although other marine environments (e.g., the intertidal zone) are also variable, hydrothermal environments are particularly unstable due to (i) the intensity of variations observed in deep-sea vents, e.g., variation of temperature from 400 to 2°C over few centimeters [3], (ii) local random mix of vent fluids with surrounding waters [4] and (iii) the relatively short lifetime of vents [5]. Because such unstable environment may be difficult to live in, deep-sea vents are often considered as harsh and stressful. For example, the vent shrimps *Rimicaris exoculata*, which usually live between 15°C and 30°C, endure sudden changes of thermal conditions due to the convection of fluids and can survive to exposure to very high temperature vent emissions [6,7].

In terms of biome characteristics, hydrothermal ecosystems are also peculiar, as they show a much higher density of individuals compared with surrounding abyssal plains. For example, populations of *R. exoculata *can reach up to 2500 individuals per square meter [8]. On the other hand, in comparison with coastal environments, hydrothermal vents are associated to a small number of species [9,10] (less than 600 species described to date in [11]). These features, along with the variable nature of the hydrothermal environment, suggest that deep-sea vent organisms may present particular genetic characteristics in relation to their peculiar adaptive abilities. Studying transposable elements (TEs) constitute an interesting way to investigate the genomic bases of such adaptive capacities. Several studies have shown that environmental variations can promote genome plasticity through transcriptional activation and mobilization of TEs (especially retrotransposons), often in response to specific stimuli such as biotic stress (e.g., pathogens) and abiotic environmental changes (e.g., temperature) [12-15]. For these reasons, TEs have a large impact on genome structure and stability, contribute in particular to variations in genome size [16-19], and are therefore considered as one of the major sources of genetic variability in eukaryotes [20-24]. The order Decapoda is a great model to investigate the genomic nature of the adaptation of hydrothermal organisms and its possible relationship with TEs. First, decapod crustaceans (shrimps, lobsters, crabs, ...) are found in various environments, with about 50 species described in hydrothermal vents [11]. Second, they exhibit great variation in genome size, from 1.07 pg (1050 Mb) in the crab *Carcinus maenas *to 40.89 pg (40000 Mb) in the shrimp *Sclerocrangon ferox *[25,26], with several species (e.g., shrimps) showing particularly large genomes that are thus liable to harbor high TE contents. Given their abundance and diversity, decapods have been greatly underrepresented in studies of TEs with only few elements described to date [27-35] including a LTR retrotransposon, GalEa1, that we have recently characterized in the hydrothermal galatheid squat lobster *Munidopsis recta *[36].

**Figure 1:**
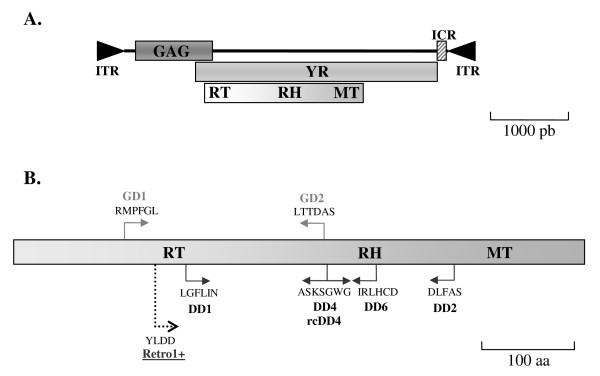
**Complete structure of *DIRS1*-like retrotransposons indicating the position of degenerate primers along the *pol *region**. **A**. Structure of the DIRS1 element identified in the slime mold *Dictyostelium discoideum*. The inverted terminal repeats (ITR) and the Internal Complementary Region (ICR) are represented by black triangles and hatched box, respectively. The three ORFs, encoding the GAG, the tyrosine recombinase (YR) and the *pol *regions (Reverse transcriptase (RT)/RnaseH (RH)/MethylTransferase (MT) domain series) correspond to shaded boxes. **B**. Positions of the degenerated primers defined in this study along the *DIRS1*-like *pol *region. Three types of primers are represented: in grey bold, the **GD **primers that correspond to conserved motifs of *Ty3/gypsy*-like retrotransposons, also shared with *DIRS1*-like elements; in black bold, the **DD **primers that correspond to conserved motifs specific to *DIRS1*-like elements and underlined, the non degenerate primer Retro1+ that spans the highly well-conserved YLDD motif that is present in both LTR retrotransposons and *DIRS*-like elements. For each primer, the orientation (arrow) and the corresponding conserved motif (uppercase) are indicated.

LTR retrotransposons (*Ty1/copia*-like, *Ty3/gypsy*-like and *Pao/Bel*-like elements) and tyrosine recombinase encoding elements (*DIRS*-like, *Ngaro*, *Viper*) constitute two major groups of retrotransposons [37-39], a class of TEs specific to eukaryotes [40]. Several phylogenetic analyses have shown that the *pol *region of *DIRS*-like elements harbor reverse transcriptase (RT) and RNase H (RH) domains closely related to those of *Ty3/gypsy*-like LTR retrotransposons [41,42]. However, these elements differ fundamentally in structure from LTR retrotransposons as they are devoid of LTR, do not encode either Proteinase and Integrase and harbor a Methyltransferase domain (Fig. 1) [39,41]. Moreover, *DIRS*-like retrotransposons replicate through a particular mechanism that involves a tyrosine recombinase [43]. Within the *DIRS*-like superfamily [38], two distinct groups have been described: *DIRS1*-like and *PAT*-like elements. These two groups differ by the nature of their termini (inverted terminal and split direct repeats, respectively [44]) and by phylogenic relationships [45].

In contrast to LTR retrotransposons that have been found in a wide continuous range of species [39,46,47], *DIRS1*-like elements have a more patchy distribution being yet reported only in a restricted number of phylogenically diverse eukaryote organisms (Table 1) [41,43,46,48-56]. Although most of the *DIRS1*-like retrotransposon identifications were based on *in silico *approaches [41,43], they have not been yet described in several well-studied phylum (e.g., mammals or plants), and are absent from model organisms such as *Saccharomyces cerevisae *and *Drosophila melanogaster*. Moreover, only few different *DIRS1*-like families have been found per species [41] in contrast to the large variety of LTR retrotransposon families [38,57-59]. In the slime mold *Dictyostelium discoideum, DIRS1*-like retrotransposons have been shown to harbor an active heat-shock promoter in their inverted terminal repeats [60,61]. Considering the high level of thermal instability in vents, the presence of such promoters could favor TE activation in hydrothermal species.

**Table 1:** *DIRS1*-like retrotransposons previously reported even in partial sequences.

**Species**	**Phylum**	**Name**	**Sequences**^1^	**Reference**
*Apis mellifera*	Insecta	AmDIRS1	[GenBank:AADG02016821]	[48]
*Arbacia punctulata*	Echinoidea	ApDIRS1	[GenBank:BK004821]	[48]
*Cotesia congregata *Bracovirus	Viruses	CcBv31.6-31.7	[GenBank:AJ632329]	[51]
*Daphnia pulex*	Branchiopoda	DpDIRS1-2	[GenBank:ACB38665, ACB38666]	Unpublished
*Danio rerio*	Teleostei	DrDIRS1-3	[GenBank:AL590134, BK001258, BK001259]	[43,49]
*Dictyostelium discoideum*	Amoebozoa	DIRS1	[GenBank:M11339]	[52]
*Gopherus agassizii*	Sauropsida	GaDIRS1	[GenBank:AC147866]	[41]
*Lytechinus variegates*	Echinoidea	LvDIRS1	[GenBank:AC131505]	[49]
*Nasonia vitripennis*	Insecta	NviDIRS1	[GenBank:NW_001820604]	Unpublished
*Nematostella vectensis*	Cnidaria	NvDIRS1	[GenBank:XP_001627585]; Repbase	[53]
*Oikopleura dioica*	Urochordata	OdDIRS1	[GenBank:AY634219]	[54]
*Oncorhynchus mykiss*	Teleostei	OmDIRS1	[GenBank:DQ156151]	Unpublished
*Phycomyces blakesleeanus*	Zygomycota	Prt1	[GenBank:Z54337]	[55]
*Rhizopus oryzae*	Zygomycota	RoDIRS1	Retrobase	[49]
*Salmo salar*	Teleostei	SsDIRS1	[GenBank:DQ156149, DQ156150]	Unpublished
*Strongylocentrotus purpuratus*	Echinoidea	SpDIRS1-4	[GenBank:BK005158]; Retrobase	[43]
*Takifugu rubripes*	Teleostei	FrDIRS1	Repbase	[56]
*Tetraodon nigroviridis*	Teleostei	TnDIRS1	[GenBank:AF442732]	[43]
*Tribolium castaneum*	Insecta	TcDIRS1	[GenBank:AY531876]	[48]
*Xenopus laevis*	Amphibia	XlDIRS1	[GenBank:BG555156]	[43]
*Xenopus tropicalis*	Amphibia	XtDIRS1-2	[GenBank:AC144974, AC145807]	[43]

Notes: -1 Accession numbers deposited in DDBJ/EMBL/GenBank databases, in Repbase database [46] or in Retrobase database [50].

Hence, studying *DIRS1*-like elements in Decapoda could allow both to better understand the role of TEs in the adaptation of species in hydrothermal ecosystems and to investigate the dynamics of these peculiar elements. We focused on ten hydrothermal decapods that represent the three major infraorders observed (6 caridean shrimps, 3 brachyuran crabs and 1 anomuran galatheid crab). We have detected *DIRS1*-like retrotransposons in all vent shrimps and in *M. recta *as well as in 8 other non-vent decapods. To our knowledge, this paper constitutes the first study based on *DIRS1*-like element distribution both in a continuous range of species within a single taxon and in species living in various ecosystems. Fifteen new *DIRS1*-like families are at all described among 15 out of the 25 species tested. These unusual elements are thus surprisingly more widely distributed among decapods than expected.

## Results

### Fishing out *DIRS1*-like pol sequences in hydrothermal shrimps

In the course of looking for *Ty3/gypsy*-like LTR retrotransposons in three hydrothermal shrimps (*Rimicaris exoculata*, *Chorocaris chacei *and *Mirocaris fortunata*) using degenerate primers GD1 and GD2 corresponding to the "RMPFGL" and "LTTDAS" conserved motifs of the RT domain (Fig. 1), we PCR-amplified additional fragments in supplement of those that correspond to *Ty3/gypsy*-like elements (data not shown). Cloning and sequencing of these additional fragments revealed that they share high sequence identity with the *pol *region of *DIRS1*-like elements at the protein level. Detailed sequence analysis showed that the fragments observed originated from either GD1/GD1 amplifications (sequences *RexAlvi1a *from *R. exoculata *and *CchAlvi1a *from *C. chacei*, [GenBank:FJ707538-FJ707539]) or from GD1/GD2 amplification with annealing of degenerate primer GD2 at a non targeted site (sequence *MfoAlvi3a *from *M. fortunata*, [GenBank:FJ707541]). Fishing out of *DIRS1*-like elements using primers designed for *Ty3/gypsy*-like retrotransposons can be explained by the close phylogenic relationships between RT domains of *Ty3/gypsy*-like and *DIRS1*-like elements [43]. Although the regions in which the primers were designed are only partly conserved between *Ty3/gypsy*-like and *DIRS1*-like elements, partial conservation may be sufficient for them to anneal on *DIRS1*-like sequences, particularly because primers are degenerate. To further confirm the presence of *DIRS1*-like elements in these species, we designed a pair of degenerate primers, DD1 and DD2, to amplify the region comprised between the "LGFLIN" and "DLFAS" motifs of the *DIRS1*-like elements *pol *region (Fig. 1). We tested these primers in *R. exoculata*. Unfortunately, they did not allow for amplifying a single band, but rather led to the amplification of numerous fragments (smear). After long and tedious optimization of the PCR conditions, we managed to obtain a major amplicon of 870 bp, which we then cloned and sequenced. Sequence analysis revealed that it corresponds actually to a DD2/DD2 amplification product, which shares similarity with *pol *region of *DIRS1*-like elements only in the last 532 bp (*RexAlvi2a*, [GenBank:FJ707540]). Due to the technical difficulties to obtain specific amplicons, we did not use this primer pair for other analyses.

To get insights on the genetic diversity of the *DIRS1*-like elements found, we aligned the partial sequences of the *pol *region that we obtained. Alignment of the 4 sequences allowed us to define 3 families. *RexAlvi1a *and *CchAlvi1a *sharing 89% nucleotide sequence identity, they were clustered into a single family that we called Alvi1 (see Additional file 1). On the other hand, *RexAlvi2a *and *MfoAlvi3a *shared too low sequence identity to either Alvi1 (< 53% and < 50% sequence identity, respectively) or to each other (71% sequence identity) to be clustered and were therefore considered as members of different families that we called Alvi2 and Alvi3.

### Screening for Alvi elements in other decapods

To date, *DIRS1*-like retrotransposons have been identified and characterized in only a restricted number of species (Table 1). To better understand the evolutionary dynamics of *DIRS1*-like elements, we investigated the diversity and the distribution of the Alvi families among several Decapoda crustacean genomes. We first analyzed the distribution of our 3 novel *DIRS1*-like families among several decapods, from both hydrothermal and non hydrothermal ecosystems. For this, we screened for Alvi families among the 25 decapods species using different combinations of primers (Table 2) including specific primers of each element and Retro1+, a primer shared by all 3 elements (see Methods and Additional file 2). Although pairs of specific primers would allow for amplification of closely related elements, we expected that the association of Retro1+ with a single specific primer would be particularly useful to extract more divergent sequences from diverse species. Cloning and sequencing of PCR products of expected size led to the identification of either the targeted elements or other *DIRS1*-like elements that belong to another family (Table 2). Sequence analysis of all the *DIRS1*-like elements found revealed that some were highly degenerate (*i.e*. they were highly divergent and harbored a large number of gaps, which did not allow for reconstructing a proper protein sequence). These elements were not included for the rest of the study (see Additional file 3). Among the other sequences, some corresponded to copies of the Alvi1-3 families, whereas some others could not be clustered to any existing families, and were considered as members of 2 new families: Alvi4 (in *Alvinocaris stactophila*) and Alvi5 (in *Alvinocaris markensis*). We thus decided to include these 2 families in our analysis. However, specific primers could not be designed for the Alvi5 family, because nucleotidic differences with the other 4 families were too spread out on the sequences. Hence, we finally analyzed the distribution of *DIRS1*-like elements in decapods using 4 families: Alvi1-4.

**Table 2:** Alvi elements detected in decapods.

		Specific primer combinations *							
		
		Alvi1 family		Alvi2 family		Alvi3 family		Alvi4 family	
								
Specimens	Environments and phylum	2 specific primers	1 specific primer ^$^	2 specific primers	1 specific primer ^$^	2 specific primers	1 specific primer ^$^	2 specific primers	1 specific primer ^$^
*Rimicaris exoculata*	hydrothermal shrimps	**RexAlvi1**	**RexAlvi1**	**RexAlvi2**	**RexAlvi2**	na	na	na	*RexAlvi1*
*Chorocaris chacei*	hydrothermal shrimps	**CchAlvi1**	**CchAlvi1**	**CchAlvi2**	A	na	*CchAlvi2*	na	A
*Mirocaris fortunata*	hydrothermal shrimps	na	**MfoAlvi1**	*MfoAlvi3*	A	**MfoAlvi3**	**MfoAlvi3**	na	*MfoAlvi3*
*Alvinocaris markensis*	hydrothermal shrimps	**AmaAlvi1**	**AmaAlvi1**	**AmaAlvi2**	A	na	*AmaAlvi5*	na	A
*Alvinocaris muricola*	hydrothermal shrimps	A	**AmuAlvi1**	**AmuAlvi2**	A	na	na	na	A
*Alvinocaris stactophila*	hydrothermal shrimps	A	**AstAlvi1**	**AstAlvi2**	A	na	*AstAlvi4*	**AstAlvi4**	**AstAlvi4**
*Segonzacia mesatlantica*	hydrothermal crabs	na	na	na	na	na	na	na	na
*Bythograea thermydron*	hydrothermal crabs	na	na	na	na	na	na	na	na
*Cyanagraea praedator*	hydrothermal crabs	na	na	na	na	na	na	na	na
*Munidopsis recta*	hydrothermal galatheid crabs	na	na	na	na	na	na	na	na
*Palaemon serratus*	intertidal shrimps	na	na	na	na	na	na	na	na
*Crangon crangon*	intertidal shrimps	na	na	na	na	na	na	na	na
Other decapods tested *	intertidal or seamount	na	na	na	-	na	na	-	na

**In black bold**: Amplicon sequences corresponding to the element targeted. Underlined: PCR amplification products in species in which the element targeted was firstly identified. *In italics*: Amplicon sequences corresponding to another element than the one targeted. A: PCR amplification product of the expected size observed but not sequenced. na: No PCR amplification of expected size. -: Not tested. * see Methods for more details. ^$ ^single specific primer associated to the Retro1+ primer.

Results, presented in Table 2, show that Alvi elements are only detected in hydrothermal shrimps. Two families (Alvi1 and Alvi2) are widely shared among these species while the two others (Alvi3 and Alvi4) are restricted to the species where they were firstly identified (*M. fortunata *and *A. stactophila*, respectively). Alvi2 elements could not be detected in *M. fortunata*. Using Alvi2 specific primers on this species we were only able to identify non-targeted elements of the Alvi3 family (*MfoAlvi3e *and *MfoAlvi3f*, [GenBank:FJ707602–FJ707603]), even using a supplementary primer (Alvi2_600-) more restrictive to Alvi2 family. Non targeted elements were also obtained when we looked for Alvi3 and Alvi4 elements (Table 2, Additional file 3), suggesting some specificity troubles especially due to the use of a single specific primer.

For all other decapods, no Alvi element could be detected even in other hydrothermal organisms or in the other closely related caridean shrimps [62]*Crangon crangon *and *Palaemon serratus*. In the hydrothermal galatheid crab *Munidopsis recta*, although no PCR amplification fragment of the expected size was obtained, we PCR-amplified an additional fragment (545 bp) when using Retro1+. Sequencing of this fragment revealed that it originated from the Retro1+/Retro1+ amplification of a new *DIRS1*-like sequence (*Ymur1a*, [GenBank:FJ707610]). It shared low sequence identity to Alvi1-5 families (< 50% sequence identity) and was therefore considered as member of a novel family that we called Ymur1.

### Diversity and phylogenetic relationships of Alvi *DIRS1*-like retrotransposons

Screening for Alvi elements among decapods led us to obtain 36 partial sequences of the *pol *region (see Additional file 3). To gain power in our investigation of the diversity of these elements, we completed these data with additional sequences. Two complementary approaches were performed: extension of the sequences by PCR walking and PCR amplification of larger internal sequences using specific primers (see Methods and Additional file 4). Seventeen new sequences were thus obtained (see Additional file 3), enabling to well-cover the *pol *region of *DIRS1*-like elements at least from the "YLDD" RT motif to the end of the RH domain (Fig. 1).

**Figure 2:**
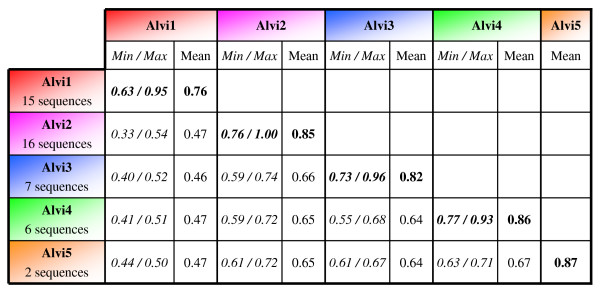
**Nucleotide sequence identity matrix of the Alvi1 to Alvi5 families**. The matrix is built using the pairwise deletion of gaps option included in the MEGA4.1 software. Results of intra-family computation are given in black bold. For each intra or inter-families observation, the mean of DNA identity is indicated as well as the lowest and highest (*Min/Max*) values represented in italics. The number of sequences included in each Alvi family is also noted.

All DNA sequences obtained were aligned using ClustalX program [63] and corresponding alignment was used to analyze the relationships between families. First, we checked the boundaries of the different families through the construction of a sequence identity matrix using the pairwise gap deletion method (see Additional file 1). A group of sequences was considered as a family if its higher intra-group divergence was lower than its inter-groups divergence, without overlap of the two distributions. The clustering obtained from sequence identity matrix validates the five families that we had previously defined (Alvi1 to Alvi5, Fig. 2). Among them, the Alvi1 copies are more diverse (from 63 to 95% sequence identity, mean: 76% sequence identity) than other families ones. The Alvi1 family is also more divergent toward the four other families (53% divergence in average between the Alvi1 copies and the others) whereas Alvi2-5 families appear to be more closely related as copies from these families differ one by one by only 35% of divergence in average.

**Figure 3:**
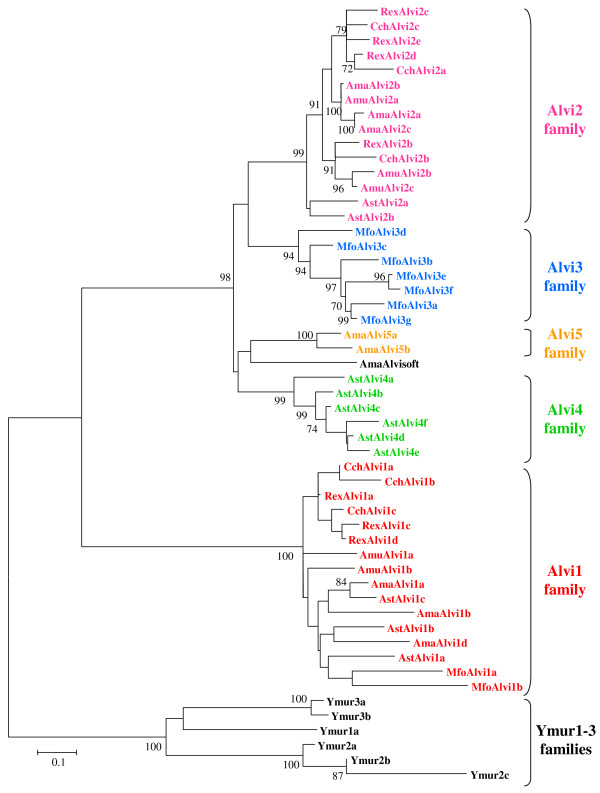
**Unrooted phenetic tree based on nucleotide *DIRS1*-like sequences identified in hydrothermal organisms**. The tree was constructed using Neighbor Joining method and pairwise deletion of gaps option included in MEGA4.1 software. Alvi1-5 families are originated from hydrothermal shrimps and Ymur1-3 elements were identified in *Munidopsis recta*. Support for individual clades was evaluated using non-parametric bootstrapping obtained from 1000 bootstrap replicates. Only bootstrap nodes value over 70% are indicated. Distances were calculated with Tamura 3 parameter model plus gamma distribution's correction for nucleotides.

Second, we analyzed the relationships of the sequences among and between the 5 families using a Neighbor Joining phylogenic analysis with pairwise gap deletion. The tree obtained (Fig. 3) confirmed that each Alvi family is well-supported with bootstrap values being greater than 94%. It also confirmed that the Alvi1 family highly diverges to the other 4 families, with strong bootstrap support (100%) of the separation into an Alvi2-5 clade, and an Alvi1 clade. Analysis of the Alvi1 subtree also revealed a lack of structuration as compared to the other families, this confirming the large sequence diversity observed with the matrix analysis. On the contrary, although relationships between families within the Alvi2-5 group can not be resolved by our tree, relationships of sequences within each family are well resolved considering most of bootstrap values being greater than 70%. It is especially the case for the Alvi2 family which sequences were obtained from various hydrothermal shrimps. Phylogenic relationships among this family also fit with those of the species [62] in which they were identified, with only few exceptions like the *AmuAlvi2a *sequence from *A. muricola *that clusters with Alvi2 sequences from *A. markensis*.

### Screening for *DIRS1*-like elements in decapods

Capacity of detection of TEs using a PCR approach depends of the choice of the primers, as shown by the identification of some elements using specific/aspecific primer pairs and not using specific/specific pairs. Hence, absence of PCR signal could be due to the divergence of the elements rather than their absence from the species analyzed. To detect such more distant *DIRS1*-like retrotransposons, we designed degenerate primers using two types of primers: "highly" degenerate and "softly" degenerate (see Methods). Although both of these approaches have flows (low copy number elements could be missed using the "highly" degenerate primers and highly divergent elements could be missed with the "softly" ones), they are complementary and should, when used in parallel, raise the detection sensitivity. Six different combinations of primers were tested (Table 3), five using "highly" degenerate primers, and one using two "softly" ones (see Methods).

**Table 3:** *DIRS1*-like retrotransposons detected among decapods.

		**Degenerate Primers Combinations**					
		
Specimens	Environments and phylum	DD1/DD6	GD1/DD6	DD4/DD6	DD1/rcDD4	GD1/rcDD4	GD1soft/rcDD4soft
*Rimicaris exoculata*	hydrothermal shrimps	**+**	**+**	**+**	**+**	**+**	**+**
*Chorocaris chacei*	hydrothermal shrimps	**+**	**+**	**+**	**+**	**+**	**+**
*Mirocaris fortunata*	hydrothermal shrimps	**+**	**+**	**+**	**+**	**+**	**+**
*Alvinocaris markensis*	hydrothermal shrimps	**+**	**+**	**+**	**+**	**+**	**AmaAlvisoft**
*Alvinocaris muricola*	hydrothermal shrimps	**+**	**+**	**+**	**+**	**+**	**+**
*Alvinocaris stactophila*	hydrothermal shrimps	**+**	**+**	**+**	**+**	**+**	**+**
*Segonzacia mesatlantica*	hydrothermal crabs	na	na	na	Na	na	na
*Bythograea thermydron*	hydrothermal crabs	na	na	na	Na	na	**-**
*Cyanagraea praedator*	hydrothermal crabs	na	na	na	Na	na	na
*Munidopsis recta*	hydrothermal galatheid crabs	*Traces*	*Traces*	na	Na	**Ymur2**	**Ymur3**
*Palaemon serratus*	intertidal shrimps	na	na	na	Na	na	**Ypase1**
*Crangon crangon*	intertidal shrimps	na	na	na	Na	na	**Ycran1**
*Xantho incisus*	intertidal crabs	na	na	na	Na	na	*Traces*
*Xantho pilipes*	intertidal crabs	na	na	na	Na	na	**Ypili1-2**
*Maja squinado*	intertidal crabs	na	na	na	Na	na	**Ymaja1**
*Carcinus maenas*	intertidal crabs	na	na	na	Na	na	na
*Necora puber*	intertidal crabs	na	na	na	Na	na	na
*Agononida laurentae*	seamount galatheid crabs	na	na	na	Na	na	**Yago1**
*Eumunida annulosa*	seamount galatheid crabs	na	na	na	Na	na	**Yannu1**
*Eumunida sternomaculata*	seamount galatheid crabs	na	na	na	Na	na	**-**
*Porcellana platycheles*	intertidal porcelain crabs	na	na	na	Na	na	na
*Pisidia longicornis*	intertidal porcelain crabs	na	na	na	Na	na	*Traces*
*Dardanus calidus*	intertidal hermit crabs	na	na	na	Na	na	na
*Nephrops norvegicus*	intertidal lobsters	na	na	na	Na	na	**Ynno1**
*Homarus gammarus*	intertidal lobsters	na	na	na	Na	na	**Yhoga1**

**In black bold**: Amplicon sequences corresponding to *DIRS1*-like element. **+**: PCR amplification products of expected size in species known to harbor *DIRS1*-like elements. *Traces*: Very low amplification of expected size. na: No Amplification of expected size. -: Artefacts.

As expected from our previous PCR results, amplification fragments were obtained in hydrothermal shrimps whatever the degenerate primer combination used (one fragment, *AmaAlvisoft*, was sequenced as control in *A. markensis*, [GenBank:FJ707542]). We have shown that *M. recta *harbors *DIRS1*-like retrotransposons (Ymur1 element). Using 'highly" degenerate primers, clear amplification fragments of the expected size were only obtained with the GD1/rcDD4 combination. Cloning and sequencing allow to obtain three additional related sequences (*Ymur2a-c*, [GenBank:FJ707543-FJ707545], > 75% sequence identity). Two others related sequences (*Ymur3a-b*, [GenBank:FJ707546–FJ707547], 93% sequence identity) were also obtained using the "softly" degenerate primer combination, one of them (*Ymur3b*) corresponding to an unexpected additional fragment about 1100 bp (GD1soft/GD1soft amplification). Sequence analysis revealed that the *DIRS1*-like sequences obtained in *M. recta *can be clustered into 3 distinct families (Ymur1-3, see Additional file 1) and phylogenic analyses indicates that Ymur1-3 families positioned themselves as a single well supported clade (bootstrap value of 100%) (Fig. 3).

**Figure 4:**
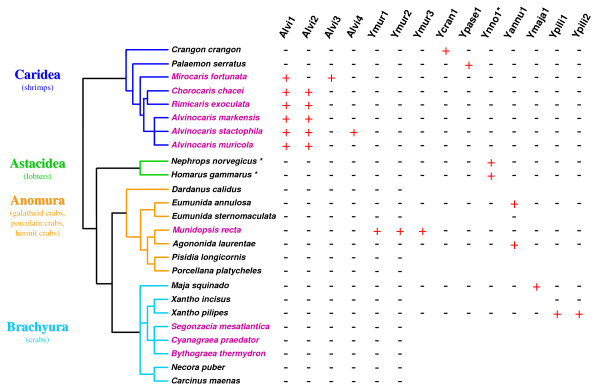
**Species distribution of the different *DIRS1*-like families**. Names of the diverse TEs analyzed are shown on top. Plus and minus signs indicate the presence (+) or absence (-) of these elements in the different species tested. Genetic relationships between species and infraorders are represented by a tree topology, reconstructed from previous studies [27,36,62,66], with hydrothermal vent species indicated in purple. Position of *Maja squinado *within Brachyura is still unresolved, this species was thus arbitrarily placed at the base of the infraorder. * The Ynno1 family of astacideans has been also detected in an EST database from another lobster species (*H. americanus*).

In other decapods, the two methods gave different results (Table 3): while using "highly" degenerate primers combinations no amplification of the expected size was obtained, the use of GD1soft/rcDD4soft led to either trace amplification or amplification fragments of the expected size. Cloning and sequencing of all single bands observed allowed us to discover new *DIRS1*-like elements ([GenBank:FJ707548–FJ707560]) in 8 new species (Fig. 4 and Additional file 3): the two coastal shrimps (*Crangon crangon *and *Palaemon serratus*), the two coastal lobsters (*Nephrops norvegicus *and *Homarus gammarus*), 2 seamount galatheid squat lobsters (*Agononida laurentae *and *Eumunida annulosa*) and 2 coastal crabs (*Xantho pilipes *and *Maja squinado*). The sequences obtained in a given species, except *X. pilipes*, cluster into a single family (> 93% sequence identity, see Additional file 1). In the crab *X. pilipes*, the two sequences obtained define two distinct families (*Ypili1a *and *Ypili2a*, 52% sequence identity). Moreover, elements of the galatheid crabs *E. annulosa *and *A. laurentae *(*Yannu1a-b *and *Yago1a*, respectively) can be clustered into a single family we called Yannu1 (> 90% sequence identity). Likewise, elements of the two lobsters *N. norvegicus *and *H. gammarus *(*Ynno1a-b *and *Yhoga1a*, respectively) clustered into the same Ynno1 family (> 82% sequence identity). We also detect in the EST databases two *DIRS1*-like sequences ([GenBank:CN853875.1, FC556055.1]) of the lobster *Homarus americanus*, that share high similarities with Ynno1 family (80% sequence identity in average). Altogether, this study led us to discover 7 new *DIRS1*-like families (see Additional file 3). All of these families diverge to Alvi1-5 and Ymur1-3 families with < 56% sequence identity (see Additional file 1).

To study their distribution, two specific primers were designed for each newly discovered *DIRS1*-like family (sequences available upon request). They were used to screen all fifteen species in which we had observed the presence of *DIRS1*-like elements (Fig. 4) as well as closely related species (same Genus). For each family, no PCR signal was obtained except in species in which the families were firstly identified. This underlines that all elements previously obtained do not result from potential cross-contaminations. Nevertheless, *DIRS1*-like retrotransposons are widely distributed among decapod crustaceans, although this distribution appears to be heterogeneous according to the phylum considered (Fig. 4). *DIRS1*-like elements are yet identified in all caridean shrimps and lobsters tested. In anomourans the distribution appears very patchy as *DIRS1*-like retrotransposons are characterized in 3 out of the 4 galatheid squat lobsters but not in porcelain crabs or hermit crab. Finally, the presence of these elements has been only detected in 2 out of the 8 brachyuran crabs tested with especially no detection in hydrothermal organisms.

## Discussion

### Methodological issues

*DIRS1*-like retrotransposons have yet been reported in a restricted number of eukaryote species (Table 1), in contrast to LTR retrotransposons that show a wide and almost continuous distribution. However, we found *DIRS1*-like elements in 15 out of the 25 decapod species studied, with 15 novel *DIRS1*-like families identified (see Additional file 3) and a particularly large diversity observed among hydrothermal shrimps (Fig. 4). To characterize transposable elements among different genomes, two approaches are usually performed: *in silico *approaches [57,58] and/or PCR-based procedures [27,28,64]. Most of previous studies aiming at identifying *DIRS1*-like retrotransposons were based on *in silico *approaches [41,43]. However, in the case of decapods, genomic sequences are scarce, with sequencing projects concerning only the creation of EST databases (e.g., *Petrolisthes cinctipes*, Joint Genome Institute). In particular, to our knowledge no large-scale genome sequencing project is ongoing. We therefore conducted several PCR-based approaches that enable to screen for partial *DIRS1*-like *pol *region simultaneously in a large number of species representing numerous decapod taxa (shrimps, crabs, galatheid crabs, porcelain crabs, hermit crabs and lobsters). Among the 73 sequences that we identified, 5 were obtained from PCR-amplified fragments which size differed from the one expected (see Additional file 3). Analyses of such additional fragments, which can be obtained for different species or using different primer combinations, provide an interesting way to identify new TEs. One issue with *DIRS*-like retrotransposons is that they are sometimes abusively grouped with *Ty3*/*gypsy*-like elements because their RT/RH domains are closely related. Distinguishing them from each other is however primordial, particularly if *DIRS*-like elements are in fact more frequent that previously supposed. For that purpose, it is important to check the partial *pol *sequence of each element for the presence of the MT domain (specific to *DIRS*-like elements) (Fig. 1) before assigning them to *DIRS*-like superfamily.

To classify our sequences into families, we used a nucleotide sequence identity matrix built using pairwise deletion method (see Additional file 1). This method presents the advantage to compute a distance for each pair of sequences, ignoring only the gaps that are involved in the comparison and therefore including the largest amount of information for the whole dataset. This method appears as the most appropriate to analyze our sequences, as our dataset is constituted of several PCR-based sequences that only partially overlap, and because many of our sequences harbor small internal indels. Finally, this approach appears efficient as it enables to clearly define the different families that we found (Fig. 2). Recently, in an attempt to find criteria for sequence-based TE classification for the annotation of TEs in large scale genome sequencing projects, the "80-80-80" rule has been proposed [38]. This rule states that two TEs belong to the same family if they share 80% (or more) sequence identity over segments longer than 80 bp representing at least 80% of the coding domains, internal domains, or terminal repeat regions. However, as noticed by the authors, this rule is more efficient on terminal repeat regions and non-coding regions than on coding regions like *pol*. This property was confirmed with our dataset, thus leading to the conclusion that, although certainly efficient for large-scale genome sequence analysis of TEs, this rule is not adapted to our PCR-based analysis.

### Distribution of Alvi families within Alvinocarididae genomes

Screenings for *DIRS1*-like elements in decapod genomes indicates that the "highly" degenerate primers have a lower detection sensitivity than the "softly" degenerate ones. Oligonucleotide sequences of "softly" degenerate primers correspond to a subset of those present in the "highly" degenerate ones (See Additional file 5). Nevertheless, the combination of "softly" degenerate primers allowed for identifying *DIRS1*-like elements in at least 15 diverse decapods whereas combinations of "highly" degenerate ones detect elements in only the 6 Alvinocarididae and *M. recta *(Table 3). Despite their weaker detection sensitivity, "highly" degenerate primers led to PCR amplifications in the six hydrothermal shrimps whatever the combinations used. We suggest that these observations may be due to a higher copy number of *DIRS1*-like retrotransposons in these genomes that could counterbalance the lower detection sensitivity of the "highly" degenerate primers.

In complement to degenerate primers, PCR screening using specific primers is a powerful approach to study the distribution of previously characterized elements, as well as for identifying novel members of closely related families. Indeed, screening for Alvi1-3 families at a small phylogenetic scale (*i.e*. within the family Alvinocarididae) led to the detection of the Alvi4 and Alvi5 families. Since all hydrothermal shrimps harbor Alvi elements, these ones likely result from the activity of *DIRS1*-like retrotransposons that were already present in the genome of the common ancestor of all Alvinocarididae species. Alvi elements may thus belong to relatively ancient lineages of *DIRS1*-like retrotransposons. They could be as old as several million years, since molecular estimates of divergence show that the vent-endemic caridean shrimp species radiated 6.7 to 11.7 million years ago [62]. This is especially true for the Alvi1 and Alvi2 families that are shared among Alvinocarididae.

Our phylogenetic analysis (Fig. 3) also shows that Alvi2 to Alvi5 families cluster into a single clade, suggesting that these families diverged from a common ancestor. The Alvi3 and Alvi4 families are each restricted to a single species and may therefore correspond to lineage-specific evolution. In *A. stactophila*, the presence of both Alvi2 and Alvi4 families suggests that this last could result from the differentiation of a subset of the ancestral family. In *M. fortunata*, which is the most distant species among vent shrimps [62], we observe the presence of Alvi3 instead of Alvi2 (Fig. 4). Two alternative evolutionary scenarios could explain this observation. If elements of the Alvi3 family were present in the Alvinocarididae ancestor, the distribution observed would result from different lineage-specific burst-like events of different Alvi families, as observed for LTR retrotransposons in rice-related species [65], leading to the amplification of Alvi3 in *M. fortunata *and this of Alvi2 in the common ancestor of other related vent shrimps. On the other hand, if the Alvi3 family was absent from the Alvinocarididae ancestor, it likely results from a lineage-specific evolution of the ancestral Alvi2 family in the *M. fortunata *lineage. In addition, *R. exoculata *and *C. chacei*, which are phylogenetically very close [62], harbor the same distribution pattern for *DIRS1*-like families (Fig. 4) with close corresponding sequences (e.g. average of 89% sequence identity within Alvi1 family). All these observations suggest that the wide distribution of *DIRS1*-like retrotransposons among Alvinocarididae is closely related to the phylogeny of species and that the emergence of new elements is linked to the independent evolutionary history of Alvi families within each host genomes. Although we cannot privilege one evolutionary scenario, all possible scenarios are in accordance with a simple vertical transmission of the different Alvi elements.

### AlDIRS1: A new clade of *DIRS1*-like retrotransposons

**Figure 5:**
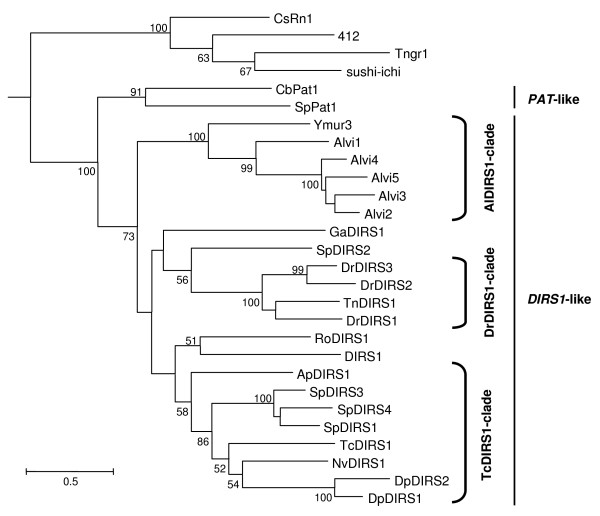
**Rooted phylogenic tree based on RT/RH amino acid sequences of *DIRS1*-like retrotransposons**. Tree performed on a core data set including representative amino acid consensus sequences of the RT/RH domains of the Ymur3 and Alvi1-5 families and 16 previously well-described *DIRS1*-like retrotransposons (see Table 1 for accession numbers). *PAT*-like sequences were used as outgroup as well as *Ty3/gypsy*-like elements according to the close phylogenic relationships of their RT/RH domains with those of *DIRS*-like retrotransposons: *Clonorchis sinensis *CsRn1, [GenBank:AAK07485]; *Drosophila melanogaster *412, [GenBank:CAA27750.1]; *Tetraodon nigroviridis *TnGr1 available on Retrobase database [50];*Takifugu rubripes *sushi-ichi, [GenBank:AAC33526]; *Caenorhabditis briggsae *CbPAT1, [GenBank:XP_001664700.1] and *Strongylocentrotus purpuratus *SpPAT1 available on Retrobase database [50]. The tree was constructed using the Maximum Likelihood method and 100 bootstrap replicates. Only nodes with bootstrap value over 50% are indicated. Distances were calculated using WAG model with gamma distribution's correction for amino acids. The two previously described clades [41,48], DrDIRS1-clade and TcDIRS1-clade, are indicated.

Previous analyses of phylogenic relationships among the *DIRS1*-like retrotransposons [41,48] highlighted two clades: the DrDIRS1 clade that groups the *DIRS1*-like elements identified in teleost fishes, and the TcDIRS1 clade that pools together elements from insects and sea urchins. To determine the relatedness of our elements with other *DIRS1*-like retrotransposons, we performed a new phylogenic analysis using the Maximum Likelihood method. The core dataset included Alvi1-5 and Ymur3 families, the only families for which we got sufficiently long sequences, as well as the recently identified elements of another crustacean species, *Daphnia pulex*, and those of the cnidarian *Nematostella vectensis*, which were never reported in phylogenic analyses to date. The resulting tree (Fig. 5) is in accordance to those previously described. The two DpDIRS1-2 elements from *D. pulex *cluster with those of other arthropods (insects) in the TcDIRS1 clade. In contrast, *DIRS1*-like elements of decapods are phylogenetically divergent from these, with the Alvi1-5 and Ymur3 families forming a distinct clade among the *DIRS1*-like superfamily that we called AlDIRS1-clade.

These results indicate the presence of amino acid signatures for decapod elements, in particular a Tryptophane (W) instead of a Phenylalanine (F) amino acid in the RT domain at position 44 on the alignment (see Additional file 6). Interestingly, this signature is also shared by the sequences that we obtained in other decapods. Therefore, we propose that the decapod *DIRS1*-like retrotransposons may belong to the AlDIRS1 clade, although further studies on the phylogenic relationships of these elements are needed to confirm this. For that purpose, two conditions should be fulfilled: to obtain additional sequences to build better amino acids consensus sequences, and to enlarge the sequences obtained for the *pol *region to improve the number of informative sites.

### Wide distribution of *DIRS1*-like elements among decapods

Our analysis of the distribution of the *DIRS1*-like retrotransposons (Fig. 4), the first as such large scale (within an order) gives clues to better understand the peculiar scattered distribution of these elements among eukaryote genomes. Three species (*M. recta*, *A. stactophila *and *A. markensis*) harbor at least 3 different *DIRS1*-like families. Such results are similar to those observed in other well-studied species such as the sea urchin *Strongylocentrotus purpuratus *(4 families, [43]) and the zebrafish *Danio rerio *(3 families, [49]). In particular, we show that *DIRS1*-like elements can be much more than just a feature of isolate species. When focusing on a given taxa (order Decapoda) the distribution of *DIRS1*-like retrotransposons (Fig. 4) appears more or less patchy, depending on the infraorder considered (as defined in [66]). Three different distribution patterns are observed: (i) presence of the elements in a large range of species (the 8 caridean and the 3 astacidean species). (ii) presence of the elements in a subpart of the infraorder (presence in 3 out of 4 galatheid crabs and absence of detection in hermit crab or porcelain crabs). (iii) presence of the elements in a restricted number of species (2 out of 8 crabs). So, Decapoda are the first example revealing that *DIRS1*-like element may be frequent, widely distributed and so probably ancient within an order.

Species distribution of *DIRS1*-like retrotransposons (Fig. 4) thus appears related to species phylogeny. Some families are widely distributed among closely related species (e.g., Alvi1-2 and Ynno1) whereas some others (e.g., Ypili1-2 and Yannu1) seem to be only scarcely shared, even among species that belong to the same Genus. Considering the environmental conditions of species, *DIRS1*-like retrotransposons are not restricted to hydrothermal species. However, their higher detection sensitivity in vent organisms (Alvinocarididae shrimps and the galatheid crab *M. recta*) suggests some peculiar features that could be related to a greatest element diversity and/or a higher number of copies. To better understand the distribution of *DIRS1*-like elements among Decapoda, the next step will be to extend our study to other infraorders (e.g. Achelata (spiny lobsters) or Dendrobranchiata (large prawn)), even to other crustaceans. Such an analysis will also allow for analyzing the vertical transmission of *DIRS1*-like elements suggested by our results. Moreover, we are currently estimating the number of copies of *DIRS1*-like retrotransposons. This will provide crucial information (i) to point out possible particularities of vent species in comparison with other decapods and (ii) to understand the dynamics of these elements within Decapoda. We also wonder whether such widespread distribution and diversity of *DIRS1*-like retrotransposons are peculiar features of Decapoda, or if similar features can be observed in other clades, for instance one known to harbor *DIRS1*-like elements (Table 1). If the widespread distribution of *DIRS1*-like retrotransposons that we observe within a taxon is a usual pattern, then the presence of one element in a given species would indicate that *DIRS1*-like retrotransposons are likely to be found in phylogenetically closely related species.

## Conclusion

Because of the small number of *DIRS1*-like elements described to date, little is known about the distribution and the evolutionary dynamics of these retrotransposons, which differ to other TEs by their peculiar tyrosine recombinase -based replication model [41]. This paper presents the first study using PCR approaches to specifically search for *DIRS1*-like retrotransposons in several related species. Fifteen new *DIRS1*-like families were identified in 15 decapod species which nearly doubles both the number of *DIRS1*-like elements described to date, and the number of species known to harbor such elements. Our study reveals a larger diversity of *DIRS1*-like retrotransposons as we distinguish a third clade (AlDIRS1-clade) in addition to the TcDIRS1 and DrDIRS1 clades previously described. Our results provide a new vision of the distribution of the *DIRS1*-like superfamily, and reveals that these elements are not as scattered as previously thought. It is the first study showing that these retrotransposons could have a widespread distribution within a large order (Decapoda) as well as a great diversity within a family (Alvinocarididae). These observations open the way to further studies on the evolutionary dynamics of these elements.

## Methods

### Biological materials

Twenty-five species of Decapoda were sampled in 3 major types of environment: deep-sea hydrothermal vents, the intertidal zone and seamounts. Vent animals were sampled during different cruises. Specimens from the South East Pacific Rise (the crabs *Bythograea thermydron *and *Cyanagraea praedator*, and the galatheid squat lobster *Munidopsis recta*) were collected during the French cruise "BIOSPEEDO" (March–May 2004) using the French "Nautile" deep-submergence vehicle operating from the N.O. "L'Atalante". Samples from the LAU Back-Arc Basin (the caridean shrimps *Alvinocaris muricola and Alvinocaris stactophila*) were collected by the DSV JASON II during the American cruise TUIM07MV (June 2005). At last, specimens from the Mid-Atlantic Ridge (the caridean shrimps *Alvinocaris markensis*, *Mirocaris fortunata*, *Rimicaris exoculata*, *Chorocaris chacei*, and the crab *Segonzacia mesatlantica*) were sampled on two vent fields (Lucky Strike or Rainbow), during the French cruise "MoMARETO" (August 2006) with the suction sampler of the ROV 'Victor 6000' operating from the R/V "Pourquoi pas ?".

For coastal decapods, 12 species (the caridean shrimps *Palaemon serratus*,*Crangon crangon*, the brachyuran crabs *Carcinus maenas*, *Maja squinado*, *Necora puber*, *Xantho incisus*,*Xantho pilipes*, the lobsters *Homarus gammarus*,*Nephrops norvegicus*, the porcelain crabs *Porcellana platycheles*, *Pisidia longicornis*, and the hermit crab *Dardanus calidus*) were collected in French Brittany.

Finally, three specimens of galatheid squat lobsters from seamounts (*Agononida laurentae*, *Eumunida annulosa*, and *Eumunida sternomaculata*) were collected south of New-Caledonia on Norfolk seamounts during the prospecting campaigns Norfolk 1 (2001, IRD Nouméa) and Norfolk 2 (2003, MUSORSTOM).

For all samples, living specimens were fixed immediately after collection, in liquid nitrogen for vent species or in 70% ethanol for the other species. They were then stored at -80°C or 4°C, respectively.

DNA from one individual per species was isolated using the CTAB method [67]. Dry DNA pellets were suspended in TE Buffer (10 mM Tris and 0.1 mM EDTA).

### Detecting *DIRS1*-like retrotransposons using degenerate primers

To look for retrotransposon *pol *fragments, we performed PCRs using several degenerate primer pairs (Fig. 1). Two primers (GD1 and GD2) were designed to amplify, in three hydrothermal shrimp species (*Chorocaris chacei*, *Mirocaris fortunata *and *Rimicaris exoculata*), conserved motifs of *Ty3/gypsy*-like retrotransposons: 'RMPFGL' and 'LTTDAS', which are also shared with *DIRS1*-like elements (see Additional file 5). Four other primers (DD1, DD2, DD4, rcDD4 (reverse complement of DD4)) were designed to amplify conserved motifs specific to the *pol *region of *DIRS1*-like retrotransposons: 'LGFLIN', 'DLFAS' and 'ASKSGWG'. Another primer, DD6, was designed on the 'IRLHCD' motif that was conserved among the elements firstly identified in this study (Alvi1-3). Detection sensitivity of *DIRS1*-like RT fragments was also improved by using, two "softly" degenerate primers related to GD1 (GD1soft) and rcDD4 (rcDD4soft). Degeneration level of these primers corresponds to 384 different sequences possible instead of the 1024 or 8192 sequences for GD1 and rcDD4, respectively. Finally, we also defined a non degenerate primer (Retro1+) conserved among *DIRS1*-like elements of Alvinocarididae and that spans the highly well-conserved YLDD motif that is present in both LTR retrotransposons and *DIRS*-like elements. All primer sequences are available in supplementary data (See Additional file 2). PCR amplifications of all primer combinations tested were performed as described in [36] and amplification products were separated on 1% agarose gels. Bands with the expected molecular weight were excised from the agarose gels, purified with SpinX column (Costar) and cloned in pGEM-T vector according to the manufacturer's recommendations (Promega, Madison, WI, USA). One to three clones were sequenced using the Abi prism automated sequencer (Genome Express) and the partial *pol *nucleotide sequences have been submitted to the GenBank database ([GenBank:FJ707538–FJ707560]).

### Extending the pol sequences

Sequences obtained with degenerate primers allowed to define several new *DIRS1*-like retrotransposons. In hydrothermal shrimps, a PCR walking approach (See Additional file 4) was then performed to extend *pol *sequences from each initial fragment, in order to study the diversity of these elements and to analyze their phylogenic relationships. PCR amplification was performed using a specific primer (sequence available upon request) designed within the fragment in combination with a degenerate primer pointing outward of either the 5' or the 3' edge of the element (see Additional file 3 for details). PCR amplifications were performed as presented above. For each walking step, one to three clones were sequenced and each individual sequence (which corresponds to part of a single genomic copy), was deposited in GenBank ([GenBank:FJ707561–FJ707569]). Each new sequence was automatically validated as an extension of the initial fragment using the Cap contig assembly program included in the BioEdit software [68] with a minimum overlap between the two sequences of 50 bp, and a minimum DNA identity of 95%.

### Analyzing the distribution of the *DIRS1*-like retrotransposons identified

Any *DIRS1*-like element that was newly identified in any species was searched in other decapods studied. Primers (sequence available upon request) were designed to be as specific as possible of each element, *i.e*. common to all sequences available for this element but unshared with the others. These specific primers were then used to PCR-screen all other species to look for well-conserved elements among species. PCR amplifications were performed for 30 cycles (94°C for 45 sec, 54°C for 1 min, and 72°C for 1 min, followed by a final extension step at 72°C for 10 min) using about 50 ng of each DNA sample, 2.5 U of Taq DNA polymerase (Invitrogen) and 10 pmol of each primer in a final volume of 25 μl. PCR products were checked by electrophoresis in a 0.8% agarose gel and those of expected size were purified using the SpinX column (Costar) then cloned in pGEM-T vector. One to three clones were sequenced (Genome Express). Corresponding sequences are available in GenBank ([GenBank: FJ707570–FJ707610]).

To investigate the distribution of *DIRS1*-like retrotransposons at a small phylogenetic scale, we focused on the Alvi elements identified within the family Alvinocarididae (hydrothermal shrimps). For that purpose, two kinds of PCR amplifications were performed, the first one using different combinations of specific primers, and the second using a specific primer in combination with the Retro1+ primer. Two or three specific primers (sequences available upon request) were designed for the Alvi1-4 families. Different combinations of primers were tested for each family (See Additional file 2) on either all 25 decapod species or on a selected subset of 12 species (*i.e*. all hydrothermal species and the two non hydrothermal caridean shrimps).

### Sequence analysis

Sequence similarity searches were performed using BLAST at the National Center for Biotechnology Information . Multiple DNA and protein sequence alignments were constructed using ClustalX [63] and manually curated using BioEdit [68]. DNA alignments were used to estimate pairwise distances and to build phenetic trees, using the pairwise deletion of gaps option of the MEGA4.1 software [69]. Pairwise distance calculation matrix was computed using p-distance model. Clustering of sequences into families was then performed in a Neighbor Joining tree [70] constructed using the Tamura 3 parameter model [71].

Representative amino acids consensus sequences of several elements were constructed by identifying the most common amino acid for each position of the alignment. Amino acid multiple alignment was checked using Gblocks [72] and ambiguously aligned sites were removed. Phylogenic analyses were then conducted using Maximum Likelihood method [73] included in the Topali2 software [74] where the best-fit model (WAG model [75] with gamma distribution) was selected. For all phylogenic analyses, support for individual clades was evaluated using non-parametric bootstrapping [76] using 1000 or 100 bootstrap replicates for nucleic acids and amino acids sequences, respectively.

## Abbreviations

bp: base pair; DNA: DesoxyriboNucleic Acid; EST: Expressed Sequence Tag; ICR: Internal Complementary Region; indel: insertion/deletion; ITR: Inversed Terminal Repeat; LINE: Long Interspersed Element; LTR: Long Terminal Repeat; Mb: Megabase; MT: MethylTransferase; ORF: Open Reading Frame; PCR: Polymerase Chain Reaction; RH: RnaseH; RT: Reverse Transcriptase; SINE: Short Interspersed Element; TE: Transposable Element; YR: Tyrosine Recombinase.

## Authors' contributions

MP carried out molecular and phylogenic analyses and participated in the design of the study. EB conceived and coordinated the study, and participated in molecular analyses. The two authors wrote and approved the final manuscript.

## Supplementary Material

Additional file 1 **Sequence identity matrix including all the nucleotide sequences acquired along the study**. Sequence identity matrix performed using the pairwise deletion of gaps option included in the Mega 4.1 software. ID: Identity. The intra-family values are indicated in colour shaded boxes. Click here for file

Additional file 2 **Retro1+ and degenerate primer sequences and specific primer combinations used for Alvi family screenings.** Click here for file

Additional file 3 **Summarization of the 73 sequences acquired in this study**. Corresponding attributes of each sequence acquired are given: TE family, length, species, PCR methodology and primers used for the identification and GenBank accession number. Click here for file

Additional file 4 **TE Walking methodology**. Summarization of the methodology used to extend the transposable element *pol *sequences. Click here for file

Additional file 5 **Retro1+ primer and degenerate primer design**. Correspondence between non specific primers used in this study and *DIRS*-like elements previously described. Mismatches are indicated in red letter. Click here for file

Additional file 6 **Amino acids sequences alignment of *DIRS1*-like retrotransposons**. Analysis including consensus sequences of the Alvi1-5 and Ymur3 families and well-described *DIRS1*-like retrotransposons from the YLDD motif of the RT domain to the ADALSR motif of the RH domain. Alignment performed using ClustalX program and corrected by Gblocks software. Amino acids signatures of all *DIRS1*-like retrotransposons are indicated in yellow. Amino acids signatures of *DIRS1*-like retrotransposons identified in hydrothermal decapods are indicated in blue. Click here for file

## References

[B1] Nyholm SV, Robidart J, Girguis PR (2008). Coupling metabolite flux to transcriptomics: insights into the molecular mechanisms underlying primary productivity by the hydrothermal vent tubeworm *Ridgeia piscesae*. Biol Bull.

[B2] Sarradin P, Caprais J, Riso R, Kerouel R, Aminot A (1999). Chemical environment of the hydrothermal mussel communities in the Lucky Strike and Menez Gwen vent fields, Mid Atlantic Ridge. Cahiers de biologie marine.

[B3] Van Dover CL, German CR, Speer KG, Parson LM, Vrijenhoek RC (2002). Evolution and biogeography of deep-sea vent and seep invertebrates. Science.

[B4] Dziak RP, Johnson HP (2002). Hydrothermal systems. Stirring the oceanic incubator. Science.

[B5] Shank TM, Fornari DJ, Von Damm KL, Lilley MD, Haymon RM, Lutz RA (1998). Temporal and spatial patterns of biological community development at nascent deep-sea hydrothermal vents (9°50'N, East Pacific Rise). Deep Sea Research Part II: Topical Studies in Oceanography.

[B6] Segonzac M, de Saint-Laurent M, Casanova B (1993). L'énigme du comportement trophique des crevettes Alvinocarididae des sites hydrothermaux de la dorsale médio-atlantique. Cahiers de biologie marine.

[B7] Ravaux J, Gaill F, Le Bris N, Sarradin P, Jollivet D, Shillito B (2003). Heat-shock response and temperature resistance in the deep-sea vent shrimp *Rimicaris exoculata*. J Exp Biol.

[B8] Desbruyères D, Segonzac M (1997). Handbook of Deep-sea Hydrothermal Vent Fauna.

[B9] Jollivet D (1996). Specific and genetic diversity at deep-sea hydrothermal vents: an overview. Biodiversity and Conservation.

[B10] Van Dover CL, Trask JL (2000). Diversity at deep-sea hydrothermal vent and intertidal mussel beds. Marine Ecology Progress Series.

[B11] Desbruyeres D, Segonzac M, Bright M (2006). Handbook of Deep-Sea Hydrothermal Vent Fauna.

[B12] Wessler SR (1996). Turned on by stress. Plant retrotransposons. Curr Biol.

[B13] Grandbastien M (1998). Activation of plant retrotransposons under stress conditions. Trends in Plant Science.

[B14] Kalendar R, Tanskanen J, Immonen S, Nevo E, Schulman AH (2000). Genome evolution of wild barley (*Hordeum spontaneum*) by BARE-1 retrotransposon dynamics in response to sharp microclimatic divergence. Proc Natl Acad Sci USA.

[B15] Melayah D, Bonnivard E, Chalhoub B, Audeon C, Grandbastien MA (2001). The mobility of the tobacco Tnt1 retrotransposon correlates with its transcriptional activation by fungal factors. Plant J.

[B16] Bennetzen JL, Kellogg EA (1997). Do Plants Have a One-Way Ticket to Genomic Obesity?. Plant Cell.

[B17] Gregory TR (2005). The C-value enigma in plants and animals: a review of parallels and an appeal for partnership. Ann Bot (Lond).

[B18] Hawkins JS, Grover CE, Wendel JF (2008). Repeated big bangs and the expanding universe: Directionality in plant genome size evolution. Plant Science.

[B19] Vitte C, Panaud O (2003). Formation of solo-LTRs through unequal homologous recombination counterbalances amplifications of LTR retrotransposons in rice *Oryza sativa *L. Mol Biol Evol.

[B20] Biémont C, Vieira C (2006). Genetics: junk DNA as an evolutionary force. Nature.

[B21] Gaut BS, Ross-Ibarra J (2008). Selection on major components of angiosperm genomes. Science.

[B22] Kazazian HH (2004). Mobile elements: drivers of genome evolution. Science.

[B23] Kidwell MG, Lisch D (1997). Transposable elements as sources of variation in animals and plants. Proc Natl Acad Sci USA.

[B24] Lagemaat LN van de, Landry J, Mager DL, Medstrand P (2003). Transposable elements in mammals promote regulatory variation and diversification of genes with specialized functions. Trends Genet.

[B25] Gregory TR Animal Genome Size Database. http://www.genomesize.com/index.php.

[B26] Rees DJ, Belzile C, Glemet H, Dufresne F (2008). Large genomes among caridean shrimp. Genome.

[B27] Bui Q, Casse N, Leignel V, Nicolas V, Chénais B (2008). Widespread occurence of mariner transposons in coastal crabs. Mol Phylogenet Evol.

[B28] Hizer SE, Tamulis WG, Robertson LM, Garcia DK (2008). Evidence of multiple retrotransposons in two litopenaeid species. Anim Genet.

[B29] Alcivar-Warren A, Meehan-Meola D, Wang Y, Guo X, Zhou L, Xiang J, Moss S, Arce S, Warren W, Xu Z, Bell K (2006). Isolation and Mapping of Telomeric Pentanucleotide (TAACC)n Repeats of the Pacific Whiteleg Shrimp, *Penaeus vannamei*, Using Fluorescence In Situ Hybridization. Marine Biotechnology.

[B30] Bui Q, Delaurière L, Casse N, Nicolas V, Laulier M, Chénais B (2007). Molecular characterization and phylogenetic position of a new mariner-like element in the coastal crab, *Pachygrapsus marmoratus*. Gene.

[B31] Casse N, Pradier E, Loiseau C, Bigot Y, Laulier M (2000). Mariner, a mobile DNA transposon in the genome of several hydrothermal invertebrates. Inter Ridge News.

[B32] Casse N, Bui QT, Nicolas V, Renault S, Bigot Y, Laulier M (2006). Species sympatry and horizontal transfers of Mariner transposons in marine crustacean genomes. Mol Phylogenet Evol.

[B33] de la Vega E, Hall MR, Wilson KJ, Reverter A, Woods RG, Degnan BM (2007). Stress-induced gene expression profiling in the black tiger shrimp *Penaeus monodon*. Physiol Genomics.

[B34] de la Vega E, Degnan BM, Hall MR, Wilson KJ (2007). Differential expression of immune-related genes and transposable elements in black tiger shrimp (*Penaeus monodon*) exposed to a range of environmental stressors. Fish Shellfish Immunol.

[B35] Halaimia-Toumi N, Casse N, Demattei MV, Renault S, Pradier E, Bigot Y, Laulier M (2004). The GC-rich transposon Bytmar1 from the deep-sea hydrothermal crab, *Bythograea thermydron*, may encode three transposase isoforms from a single ORF. J Mol Evol.

[B36] Terrat Y, Bonnivard E, Higuet D (2008). GalEa retrotransposons from galatheid squat lobsters (Decapoda, Anomura) define a new clade of Ty1/copia-like elements restricted to aquatic species. Mol Genet Genomics.

[B37] Lorenzi HA, Robledo G, Levin MJ (2006). The VIPER elements of trypanosomes constitute a novel group of tyrosine recombinase-enconding retrotransposons. Mol Biochem Parasitol.

[B38] Wicker T, Sabot F, Hua-Van A, Bennetzen JL, Capy P, Chalhoub B, Flavell A, Leroy P, Morgante M, Panaud O, Paux E, SanMiguel P, Schulman AH (2007). A unified classification system for eukaryotic transposable elements. Nat Rev Genet.

[B39] Eickbush TH, Jamburuthugoda VK (2008). The diversity of retrotransposons and the properties of their reverse transcriptases. Virus Res.

[B40] Capy P, Bazin C, Higuet D, Langin T (1997). Dynamics and Evolution of Transposable Elements.

[B41] Poulter RTM, Goodwin TJD (2005). DIRS-1 and the other tyrosine recombinase retrotransposons. Cytogenet Genome Res.

[B42] Chang GS, Hong Y, Ko KD, Bhardwaj G, Holmes EC, Patterson RL, van Rossum DB (2008). Phylogenetic profiles reveal evolutionary relationships within the "twilight zone" of sequence similarity. Proc Natl Acad Sci USA.

[B43] Goodwin TJ, Poulter RT (2001). The DIRS1 group of retrotransposons. Mol Biol Evol.

[B44] de Chastonay Y, Felder H, Link C, Aeby P, Tobler H, Muller F (1992). Unusual features of the retroid element PAT from the nematode *Panagrellus redivivus*. Nucl Acids Res.

[B45] Duncan L, Bouckaert K, Yeh F, Kirk DL (2002). kangaroo, a mobile element from *Volvox carteri*, is a member of a newly recognized third class of retrotransposons. Genetics.

[B46] Jurka J, Kapitonov VV, Pavlicek A, Klonowski P, Kohany O, Walichiewicz J Repbase Update – GIRI. http://www.girinst.org/repbase/update/index.html.

[B47] Lloréns C, Futami R, Bezemer D, Moya A The Gypsy Database (GyDB) of Mobile Genetic Elements. http://gydb.uv.es/index.php.

[B48] Goodwin TJD, Poulter RTM, Lorenzen MD, Beeman RW (2004). DIRS retroelements in arthropods: identification of the recently active TcDirs1 element in the red flour beetle *Tribolium castaneum*. Mol Genet Genomics.

[B49] Goodwin TJD, Poulter RTM (2004). A new group of tyrosine recombinase-encoding retrotransposons. Mol Biol Evol.

[B50] Poulter RTM Retrobase. http://biocadmin.otago.ac.nz/fmi/xsl/retrobase/home.xsl.

[B51] Drezen J, Bézier A, Lesobre J, Huguet E, Cattolico L, Periguet G, Dupuy C (2006). The few virus-like genes of *Cotesia congregata *bracovirus. Arch Insect Biochem Physiol.

[B52] Zuker C, Cappello J, Chisholm RL, Lodish HF (1983). A repetitive *Dictyostelium *gene family that is induced during differentiation and by heat shock. Cell.

[B53] Putnam NH, Srivastava M, Hellsten U, Dirks B, Chapman J, Salamov A, Terry A, Shapiro H, Lindquist E, Kapitonov VV, Jurka J, Genikhovich G, Grigoriev IV, Lucas SM, Steele RE, Finnerty JR, Technau U, Martindale MQ, Rokhsar DS (2007). Sea anemone genome reveals ancestral eumetazoan gene repertoire and genomic organization. Science.

[B54] Volff J, Lehrach H, Reinhardt R, Chourrout D (2004). Retroelement dynamics and a novel type of chordate retrovirus-like element in the miniature genome of the tunicate *Oikopleura dioica*. Mol Biol Evol.

[B55] Ruiz-Pérez VL, Murillo FJ, Torres-Martínez S (1996). Prt1, an unusual retrotransposon-like sequence in the fungus *Phycomyces blakesleeanus*. Mol Gen Genet.

[B56] Aparicio S, Chapman J, Stupka E, Putnam N, Chia J, Dehal P, Christoffels A, Rash S, Hoon S, Smit A, Gelpke MD, Roach J, Oh T, Ho IY, Wong M, Detter C, Verhoef F, Predki P, Tay A, Lucas S, Richardson P, Smith SF, Clark MS, Edwards YJK, Doggett N, Zharkikh A, Tavtigian SV, Pruss D, Barnstead M, Evans C, Baden H, Powell J, Glusman G, Rowen L, Hood L, Tan YH, Elgar G, Hawkins T, Venkatesh B, Rokhsar D, Brenner S (2002). Whole-genome shotgun assembly and analysis of the genome of *Fugu rubripes*. Science.

[B57] Wicker T, Keller B (2007). Genome-wide comparative analysis of copia retrotransposons in Triticeae, rice, and *Arabidopsis *reveals conserved ancient evolutionary lineages and distinct dynamics of individual copia families. Genome Res.

[B58] Wang H, Liu J (2008). LTR retrotransposon landscape in *Medicago truncatula*: more rapid removal than in rice. BMC Genomics.

[B59] Rho M, Choi J, Kim S, Lynch M, Tang H (2007). De novo identification of LTR retrotransposons in eukaryotic genomes. BMC Genomics.

[B60] Zuker C, Cappello J, Lodish HF, George P, Chung S (1984). *Dictyostelium *transposable element DIRS-1 has 350-base-pair inverted terminal repeats that contain a heat shock promoter. Proc Natl Acad Sci USA.

[B61] Cappello J, Zuker C, Lodish HF (1984). Repetitive *Dictyostelium *heat-shock promotor functions in *Saccharomyces cerevisiae*. Mol Cell Biol.

[B62] Shank TM, Black MB, Halanych KM, Lutz RA, Vrijenhoek RC (1999). Miocene radiation of deep-sea hydrothermal vent shrimp (Caridea: Bresiliidae): evidence from mitochondrial cytochrome oxidase subunit I. Mol Phylogenet Evol.

[B63] Thompson JD, Gibson TJ, Plewniak F, Jeanmougin F, Higgins DG (1997). The CLUSTAL_X windows interface: flexible strategies for multiple sequence alignment aided by quality analysis tools. Nucleic Acids Res.

[B64] Flavell AJ, Smith DB, Kumar A (1992). Extreme heterogeneity of Ty1-copia group retrotransposons in plants. Mol Gen Genet.

[B65] Panaud O, Vitte C, Hivert J, Muzlak S, Talag J, Brar D, Sarr A (2002). Characterization of transposable elements in the genome of rice (*Oryza sativa *L.) using Representational Difference Analysis (RDA). Mol Genet Genomics.

[B66] Tsang LM, Ma KY, Ahyong ST, Chan T, Chu KH (2008). Phylogeny of Decapoda using two nuclear protein-coding genes: origin and evolution of the Reptantia. Mol Phylogenet Evol.

[B67] Ishaq M, Wolf B, Ritter C (1990). Large-scale isolation of plasmid DNA using cetyltrimethylammonium bromide. Biotechniques.

[B68] Hall TA (1999). BioEdit: a user-friendly biological sequence alignment editor and analysis program for Windows 95/98/NT. Nucleic acids symposium series.

[B69] Tamura K, Dudley J, Nei M, Kumar S (2007). MEGA4: Molecular Evolutionary Genetics Analysis (MEGA) software version 4.0. Mol Biol Evol.

[B70] Saitou N, Nei M (1987). The neighbor-joining method: a new method for reconstructing phylogenetic trees. Mol Biol Evol.

[B71] Tamura K, Kumar S (2002). Evolutionary distance estimation under heterogeneous substitution pattern among lineages. Mol Biol Evol.

[B72] Castresana J (2000). Selection of conserved blocks from multiple alignments for their use in phylogenetic analysis. Mol Biol Evol.

[B73] Guindon S, Gascuel O (2003). A simple, fast, and accurate algorithm to estimate large phylogenies by maximum likelihood. Syst Biol.

[B74] Milne I, Wright F, Rowe G, Marshall DF, Husmeier D, McGuire G (2004). TOPALi: software for automatic identification of recombinant sequences within DNA multiple alignments. Bioinformatics.

[B75] Whelan S, Goldman N (2001). A general empirical model of protein evolution derived from multiple protein families using a maximum-likelihood approach. Mol Biol Evol.

[B76] Felsenstein J (1985). Confidence limits on phylogenies: an approach using the bootstrap. Evolution.

