# Supplementary figures and images for: DIRS1-like retrotransposons are widely distributed among Decapoda and are particularly present in hydrothermal vent organisms
Source: BMC Evol Biol. 2009 Apr 28;9:86. doi: 10.1186/1471-2148-9-86 (PMC2685390; doi:10.1186/1471-2148-9-86)

## A) TE walking

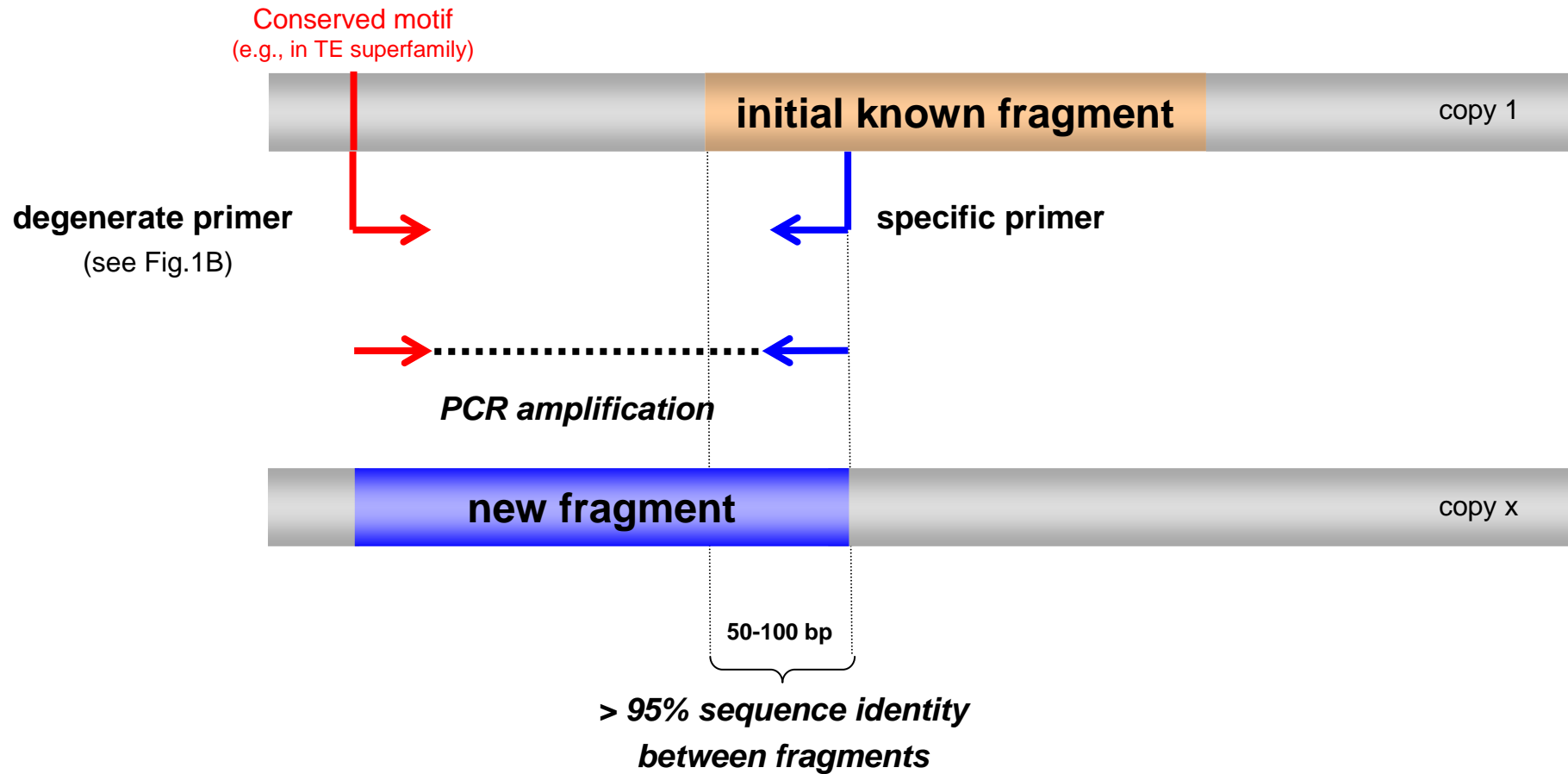

## B) Chimeric amino acid sequence reconstruction

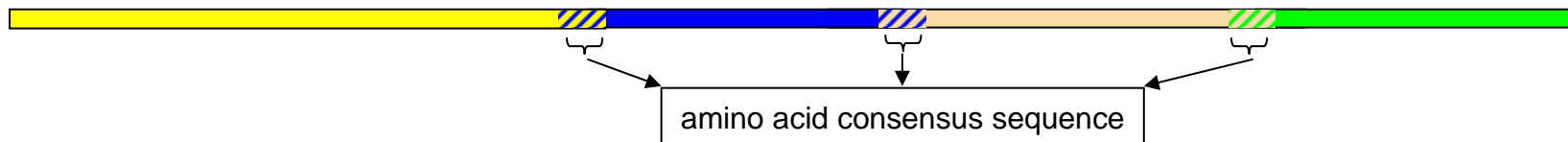

Supplement: Additional file 4 — TE Walking methodology. Summarization of the methodology used to extend the transposable element pol sequences. [file 1471-2148-9-86-S4.pdf]
